# Glycogen synthase kinase homolog Rim11 regulates lipid synthesis through the phosphorylation of Pah1 phosphatidate phosphatase in yeast

**DOI:** 10.1016/j.jbc.2022.102221

**Published:** 2022-07-01

**Authors:** Shoily Khondker, Joanna M. Kwiatek, Gil-Soo Han, George M. Carman

**Affiliations:** Department of Food Science and the Rutgers Center for Lipid Research, New Jersey Institute for Food, Nutrition, and Health, Rutgers University, New Brunswick, New Jersey, USA

**Keywords:** phosphatidate, diacylglycerol, triacylglycerol, phosphatidate phosphatase, Pah1, lipin, glycogen synthase kinase, Rim11, phosphorylation, yeast, DAG, diacylglycerol, ER, endoplasmic reticulum, GSK3β, glycogen synthase kinase-3β, LC-MS/MS, liquid chromatography/tandem mass spectrometry, PA, phosphatidate, PAP, phosphatidate phosphatase, SC, synthetic complete, TAG, triacylglycerol

## Abstract

Pah1 phosphatidate (PA) phosphatase plays a major role in triacylglycerol synthesis in *Saccharomyces cerevisiae* by producing its precursor diacylglycerol and concurrently regulates *de novo* phospholipid synthesis by consuming its precursor PA. The function of Pah1 requires its membrane localization, which is controlled by its phosphorylation state. Pah1 is dephosphorylated by the Nem1-Spo7 protein phosphatase, whereas its phosphorylation occurs by multiple known and unknown protein kinases. In this work, we show that Rim11, a yeast homolog of mammalian glycogen synthase kinase-3β, is a protein kinase that phosphorylates Pah1 on serine (Ser12, Ser602, and Ser818) and threonine (Thr163, Thr164, Thr522) residues. Enzymological characterization of Rim11 showed that its *K*_m_ for Pah1 (0.4 μM) is similar to those of other Pah1-phosphorylating protein kinases, but its *K*_m_ for ATP (30 μM) is significantly higher than those of these same kinases. Furthermore, we demonstrate Rim11 phosphorylation of Pah1 does not require substrate prephosphorylation but was increased ∼2-fold upon its prephosphorylation by the Pho85-Pho80 protein kinase. In addition, we show Rim11-phosphorylated Pah1 was a substrate for dephosphorylation by Nem1-Spo7. Finally, we demonstrate the Rim11 phosphorylation of Pah1 exerted an inhibitory effect on its PA phosphatase activity by reduction of its catalytic efficiency. Mutational analysis of the major phosphorylation sites (Thr163, Thr164, and Ser602) indicated that Rim11-mediated phosphorylation at these sites was required to ensure Nem1-Spo7-dependent localization of the enzyme to the membrane. Overall, these findings advance our understanding of the phosphorylation-mediated regulation of Pah1 function in lipid synthesis.

In the yeast *Saccharomyces cerevisiae*, Pah1 is a Mg^2+^-dependent phosphatidate (PA) phosphatase (PAP) catalyzing the conversion of PA to diacylglycerol (DAG) (Fig. 1) (1, 2, 3). The enzyme provides the immediate precursor of triacylglycerol (TAG) and at the same time controls the level of PA that is required for the *de novo* synthesis of membrane phospholipids (Fig. 1) (4, 5, 6, 7, 8, 9). The PAP-derived DAG is also used for the synthesis of phosphatidylcholine and phosphatidylethanolamine by the CDP-choline and CDP-ethanolamine branches, respectively, of the Kennedy pathway when cells are exogenously supplied with choline and ethanolamine (8, 10). As expected, Pah1 expression and PAP activity are increased as yeast cells progress to the stationary phase when TAG synthesis is more active than phospholipid synthesis (2, 11, 12, 13).

**Figure 1:**
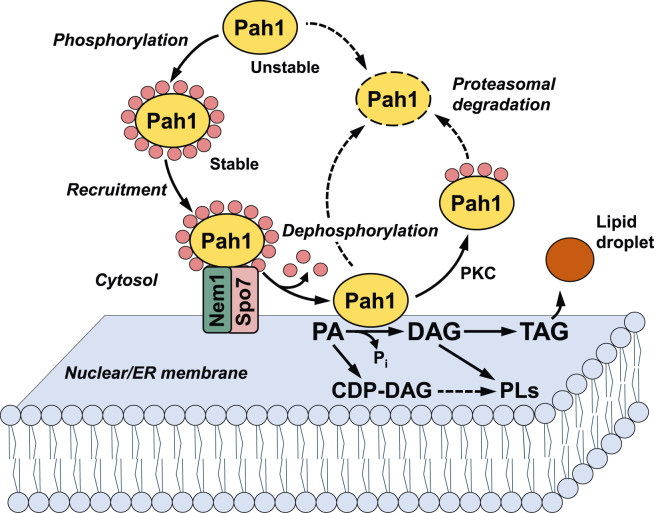
**Model for localization and regulation of Pah1 by phosphorylation and dephosphorylation.** Unphosphorylated Pah1 in the cytosol is unstable but stabilized through its phosphorylation by multiple protein kinases. The phosphorylated Pah1 translocates to the nuclear/ER membrane through its recruitment and dephosphorylation by the Nem1-Spo7 protein phosphatase complex. Dephosphorylated Pah1, which is associated with the membrane, catalyzes the conversion of phosphatidate (PA) to diacylglycerol (DAG) for the synthesis of triacylglycerol (TAG) stored in lipid droplets. Dephosphorylated Pah1 or *PKC*-phosphorylated Pah1 lacking phosphorylation by Pho85-Pho80/Cdc28-cyclin B is susceptible to degradation by the proteasome (indicated by the *dashed line arrows* and *ellipse*). The phosphatidate phosphatase substrate PA is also converted to CDP-DAG for the synthesis of the membrane phospholipids phosphatidylserine, phosphatidylethanolamine, phosphatidylcholine, phosphatidylinositol, phosphatidylglycerol, and cardiolipin. The phosphatidate phosphatase product DAG is also used for the synthesis of phosphatidylcholine and phosphatidylethanolamine *via* the Kennedy pathway. Further details for the yeast phospholipid synthetic pathways can be found elsewhere (8, 9, 10, 107, 108).

By controlling the PA/DAG level, Pah1 PAP regulates lipid synthesis gene expression *via* the Opi1/Ino2-Ino4 (Henry) regulatory circuit (8, 14, 15), phospholipid synthesis (11), nuclear/endoplasmic reticulum (ER) membrane growth (14), and lipid droplet formation (16). Pah1 PAP is also required for growth on nonfermentable carbon sources (2, 17), vacuole fusion (18), cell wall integrity (19, 20), autophagy induction (21), and resistance to stresses caused by fatty acids (12), oxidizing agents (22), heat (2, 14, 23, 24), and cold (25). In fact, mutants lacking Pah1 PAP activity have a shortened chronological life span (22) and exhibit apoptotic cell death in the stationary phase (12). Many *pah1*Δ phenotypes are linked to elevated PA content and require Dgk1 DAG kinase activity (12, 16, 22, 24). In mice and humans, the loss of lipin 1 PAP is associated with the lipid-based syndromes such as lipodystrophy, peripheral neuropathy, and rhabdomyolysis (26, 27, 28, 29, 30, 31).

The cellular function of Pah1 is primarily regulated by its localization. Following its expression that is controlled by nutrients at the transcriptional level (11, 32), Pah1 is phosphorylated on the serine and threonine residues (33, 34, 35, 36, 37, 38, 39, 40, 41, 42, 43, 44, 45, 46) by many protein kinases (47, 48, 49, 50, 51, 52). The phosphorylated form of Pah1 is stable in the cytosol but nonfunctional due to the lack of its association with the PA-containing membrane. In order to be functional, the phosphorylated form undergoes dephosphorylation by the Nem1 (catalytic)-Spo7 (regulatory) protein phosphatase in the nuclear/ER membrane (14, 34, 47, 48, 49, 53, 54, 55, 56, 57). Following its dephosphorylation, Pah1 hops onto, associates with the membrane *via* its N-terminal amphipathic helix, and dephosphorylates PA to produce DAG, which is then acylated to form TAG that is stored in lipid droplets (Fig. 1). The functional Pah1 may subsequently scoot on the membrane for additional rounds of catalysis (58).

The two conserved domains (N-LIP and haloacid dehalogenase–like) of Pah1 (2, 28) are required for its PAP activity (17, 59); Asp398 and Asp400 in the D*X*D*X*(T/V) motif are essential for catalytic activity (17, 28, 60) (Fig. 2). In addition, a conserved tryptophan residue (Trp637) (59) located in the intrinsically disordered region plays a role in Pah1 phosphorylation, its dephosphorylation-mediated translocation to the membrane, and its function in TAG synthesis (59, 61). Trp637, however, is not essential for the catalytic function of the enzyme (59, 61). According to the AlphaFold model of Pah1 (62), Trp637 and the catalytic residues (*i.e.*, Asp398 and Asp400) lie in the same plane (Fig. 2*B*), suggesting the importance of their alignment on the membrane surface for the enzyme to recognize its substrate (61). In addition, the N-LIP and haloacid dehalogenase–like domains are shown to closely interact, and the phosphorylation sites primarily reside within the intrinsically disordered regions (Fig. 2*B*).

**Figure 2:**
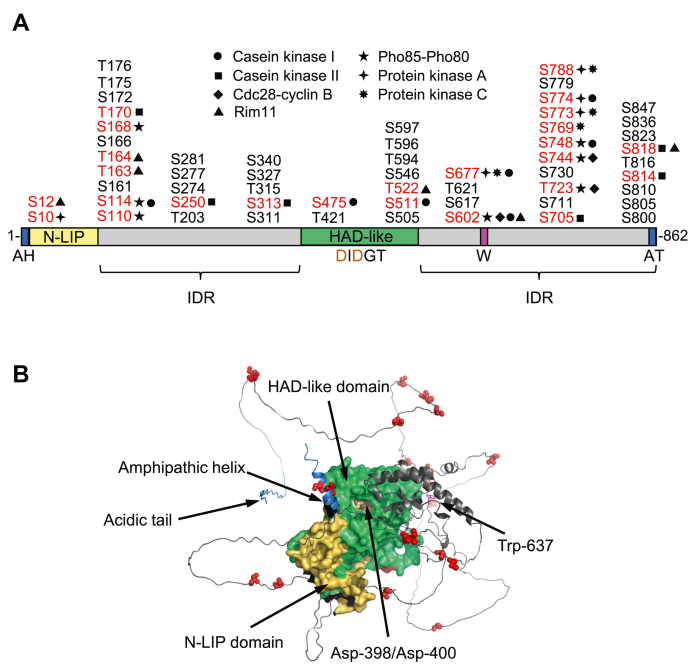
**Pah1 domains, regions, and phosphorylation sites.***A*, the schematic shows the positions of the N-terminal amphipathic helix (*AH*), the conserved N-LIP and haloacid dehalogenase–like catalytic domains, the conserved tryptophan (*W*) residue, the C-terminal acidic tail (*AT*), and the intrinsically disordered region (*IDR*). The serine (*S*) and threonine (*T*) residues known to be phosphorylated (33, 34, 35, 36, 37, 38, 39, 40, 41, 42, 43, 44, 47, 48, 49, 50, 51, 52, 61) are grouped at their approximate regions in the protein. The sites phosphorylated by casein kinase I (52), casein kinase II (51), Cdc28-cyclin B (48), Pho85-Pho80 (47), protein kinase A (49), protein kinase C (50), and Rim11 (this study) are indicated in *red*. *B*, the AlphaFold (62) structure prediction of Pah1 was visualized with the PyMol program. The phosphorylation sites with known responsible protein kinases are marked in *red*.

Pah1 is easily degraded by the 20S proteasome when it is dephosphorylated (or unphosphorylated) but stable against the proteasomal degradation when it is phosphorylated (63, 64). An exception to the protective effect of phosphorylation is shown by protein kinase C whose activity on Pah1 lacking prephosphorylation by Pho85-Pho80 stimulates its proteasomal degradation (50). The Nem1 and Spo7 subunits, which form a phosphatase complex to regulate the phosphorylation state of Pah1, are themselves subject to phosphorylation (44, 65, 66, 67, 68), adding additional control of Pah1 function by phosphorylation. For Pah1, phosphorylation and dephosphorylation are major posttranslational modifications to regulate its cellular function in lipid synthesis.

The sites of Pah1 phosphorylation are primarily located in the intrinsically disordered regions between the two conserved domains and at the C-terminal region (64) (Fig. 2*A*). Many of those sites have been identified as target residues for protein kinases such as Cdc28-cyclin B (48), Pho85-Pho80 (47), protein kinase A (49), protein kinase C (50), casein kinase I (52), and casein kinase II (51) (Fig. 2*A*). The protein kinase–target site relationship is important to understand the role of a specific phosphorylation in the regulation of Pah1 for its location, PAP activity, and protein stability (69). In a global analysis of protein kinase substrates, Pah1 has been shown to be phosphorylated *in vitro* by Rim11 (70), the yeast homolog of mammalian glycogen synthase kinase-3β (GSK3β) (71). Rim11 has previously been studied in diploid cells for its role in promoting entry into meiosis by phosphorylating the components of a transcriptional activator complex consisting of Ime1 and Ume6 (72, 73, 74). In this work, we showed that the Rim11 phosphorylation of Pah1 is involved in the regulation of its function by the Nem1-Spo7 protein phosphatase. Pah1 is phosphorylated by the protein kinase on the serine and threonine residues, and its phosphorylation is inhibitory on PAP activity by a mechanism that reduces catalytic activity. This work advances the understanding of Pah1 regulation by phosphorylation and identifies a novel physiological role of the Rim11 protein kinase in the regulation of lipid synthesis.

## Results

### Purification of Rim11

**Figure 3:**
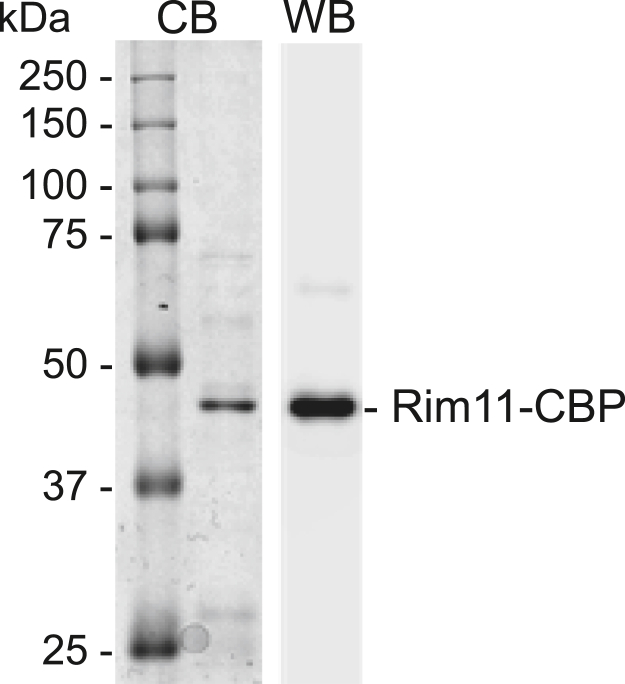
**Purification of Rim11.** Purified calmodulin binding peptide (*CBP*)-tagged Rim11 (200 ng) was subjected to SDS-PAGE (10% polyacrylamide gel) (*left*) and stained with Coomassie blue (*CB*). The purified enzyme was also subjected to Western blot (*WB*) analysis using anti-calmodulin binding peptide antibody (*right*). The positions of Rim11 and molecular mass standards are indicated.

Previous studies have shown that Rim11 is subject to autophosphorylation at Tyr199, which is required for activation of Rim11 protein kinase activity (71, 75). Owing to this requirement, we purified Rim11 from yeast since it would contain endogenous phosphorylation. The TAP-tagged Rim11 was purified by affinity chromatography with IgG-Sepharose and then digested with tobacco etch virus protease to remove the protein A tag. The protein A–free Rim11, which retains a calmodulin binding peptide tag, was then separated from the protease by anion exchange chromatography. The purified protein was nearly homogenous in the SDS-PAGE analysis (Fig. 3). The fusion protein was detected by Western blotting with anti-calmodulin binding peptide antibody (Fig. 3). In addition, the identity of Rim11 was confirmed by liquid chromatography/tandem mass spectrometry (LC-MS/MS) analysis of the peptides (38% coverage of the protein) derived from trypsin digestion of the fusion protein (Table S1).

### Pah1 is a *bona fide* substrate of Rim11 with its phosphorylation on serine and threonine residues

**Figure 4:**
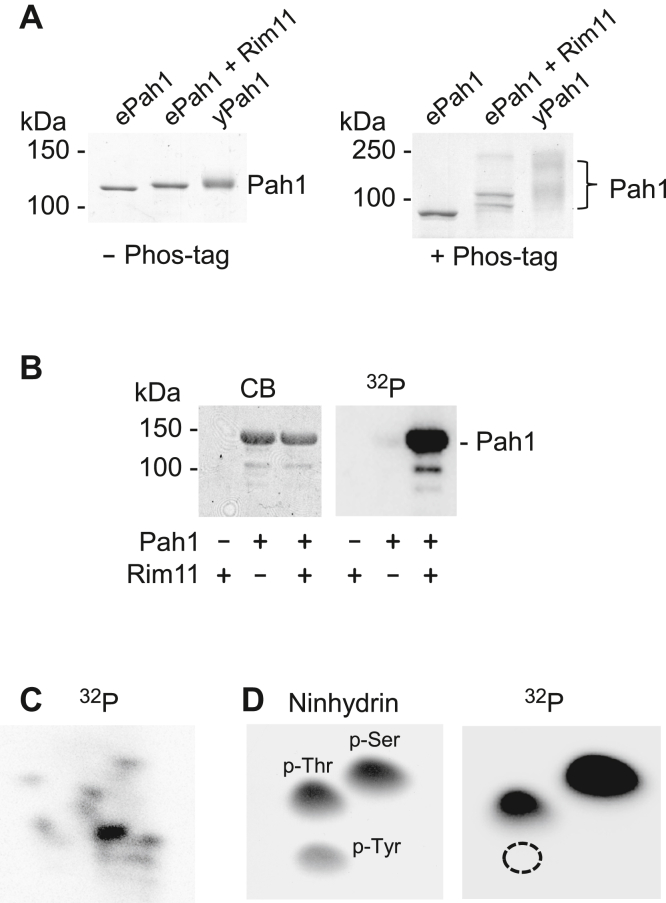
**Phosphorylation of Pah1 by Rim11 on serine and threonine residues.***A*, Pah1 (0.5 μg) expressed in *E. coli* (*ePah1*) and yeast (*yPah1*), and the *E. coli*-expressed protein phosphorylated by Rim11 (*ePah1 + Rim11*) were subjected to SDS-PAGE using 6% polyacrylamide gels in the absence (*left*) and presence (*right*) of 20 μM Phos-tag and 100 μM MnCl_2_. The resolved proteins were stained with Coomassie blue, and their electrophoretic migration is indicated with molecular mass standards. *B*, *E. coli*-expressed Pah1 (1 μg) was incubated for 1 h at 30 °C in the absence (-) and presence (+) of 0.2 μg Rim11 and 100 μM [γ-^32^P]ATP (3000 cpm/pmol). The reaction mixtures were resolved by SDS-PAGE (10% polyacrylamide gel) and subjected to phosphorimaging, followed by protein staining with Coomassie blue (*CB*). *C*, ^32^P-labeled Pah1 was digested with L-1-tosylamido-2-phenylethyl chloromethyl ketone-treated trypsin and separated on a cellulose TLC plate by electrophoresis (from *left* to *right*) in the first dimension and by chromatography (from *bottom* to *top*) in the second dimension, and the radioactive phosphopeptides were visualized by phosphorimaging. *D*, ^32^P-labeled Pah1 was hydrolyzed by 6 N HCl for 3 h at 100 °C. The acid hydrolysate was resolved on a cellulose TLC plate by two-dimensional electrophoresis and subjected to phosphorimaging (*right*). The phosphoamino acid standards phosphoserine (*p-Ser*), phosphothreonine (*p-Thr*), and phosphotyrosine (*p-Tyr*) were visualized by ninhydrin staining (*left*). The data shown in all panels are representative of three experiments.

To examine Pah1 phosphorylation by Rim11, we utilized the protein purified from its heterologous expression in *Escherichia coli*. By using the *E. coli*-expressed Pah1, we could examine its phosphorylation in the absence of the endogenous phosphorylation in yeast (34, 61). We first examined the Rim11 phosphorylation of Pah1 by its electrophoretic mobility in SDS-PAGE. Compared with unphosphorylated Pah1 (Fig. 4*A* *left, ePah1*), its phosphorylated form (Fig. 4*A* *left, ePah1 + Rim11*) showed a decrease in electrophoretic mobility. To better determine the mobility shift, Pah1 was resolved in the gel containing Phos-tag, a molecule that traps phosphorylated proteins. In the Phos-tag gel, unphosphorylated Pah1 migrated as a single discrete band (Fig. 4*A* *right, ePah1*), whereas the protein phosphorylated by Rim11 showed multiple bands of slower migration (Fig. 4*A* *right, ePah1 + Rim11*), indicating heterogeneous phosphorylation of the protein. Compared with the Rim11-phosphorylated Pah1, the protein phosphorylated endogenously in yeast showed more bands of slower electrophoretic mobility (Fig. 4*A* *left and right, yPah1*), indicating that it is highly and heterogeneously phosphorylated in the cell.

Although the electrophoretic mobility is useful to assess the phosphorylation state of Pah1, it is not as sensitive and quantitative as enzyme assays (48) that measure the transfer of phosphate from ATP to the protein. Accordingly, the Rim11 phosphorylation of Pah1 was analyzed in subsequent experiments by following the incorporation of the radioactive phosphate from [γ-^32^P]ATP into the protein. In the assay, the ^32^P-labeled Pah1 was resolved from [γ-^32^P]ATP by SDS-PAGE and then quantified by phosphorimaging (Fig. 4*B*). Phosphopeptide mapping of the ^32^P-labeled Pah1 indicated that its phosphorylation by Rim11 occurs on multiple sites (Fig. 4*C*), and phosphoamino acid analysis showed that its phosphorylation occurs on the serine and threonine residues with the former being a major target site (Fig. 4*D, right*).

**Figure 5:**
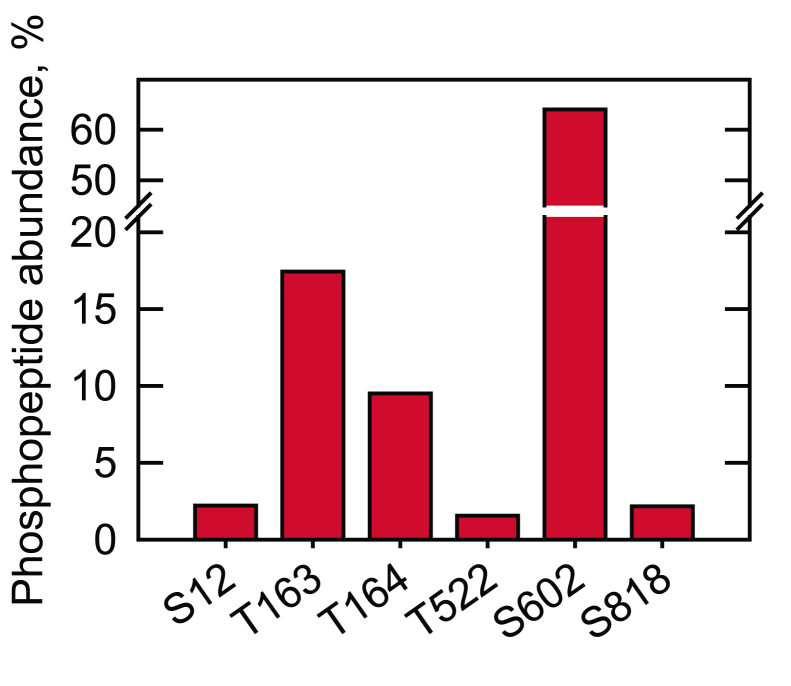
**Identification of Pah1 sites phosphorylated by Rim11.** The *E. coli*-expressed Pah1 was phosphorylated by Rim11 and resolved by SDS-PAGE. The phosphorylated Pah1 was extracted from the gel, reduced, and alkylated, followed by digestion with trypsin, chymotrypsin, or Glu-C; the resulting peptides were analyzed as described in “Experimental procedures.” Database search results from each digest were combined, and the abundance of phosphopeptides containing the indicated phosphorylation sites was estimated from intensities reported by Proteome Discoverer and expressed as a percentage of the intensities of all phosphopeptides identified for the protein (Table S2). Shown are only the phosphorylation sites that are confidently assigned at ≥ 1% of the total phosphopeptide abundances.

The phosphorylation sites of Pah1 were identified by LC-MS/MS analysis of phosphopeptides derived from its digestion with trypsin, chymotrypsin, and Glu-C (Fig. 5 and Table S2). Based on the abundance of the phosphopeptides, Ser602 (64%), Thr163 (17.5%), and Thr164 (9.5%) are the major sites of phosphorylation by Rim11 (Fig. 5). In contrast, the minor phosphorylation sites included Ser12 (2%), Thr522 (1.5%), and Ser818 (2%). Overall, phosphorylation on the serine residue accounted for ∼ 70% of the total phosphorylation, which is also indicated by the phosphoamino acid analysis of phosphorylated Pah1 (Fig. 4*D, right*).

**Figure 6:**
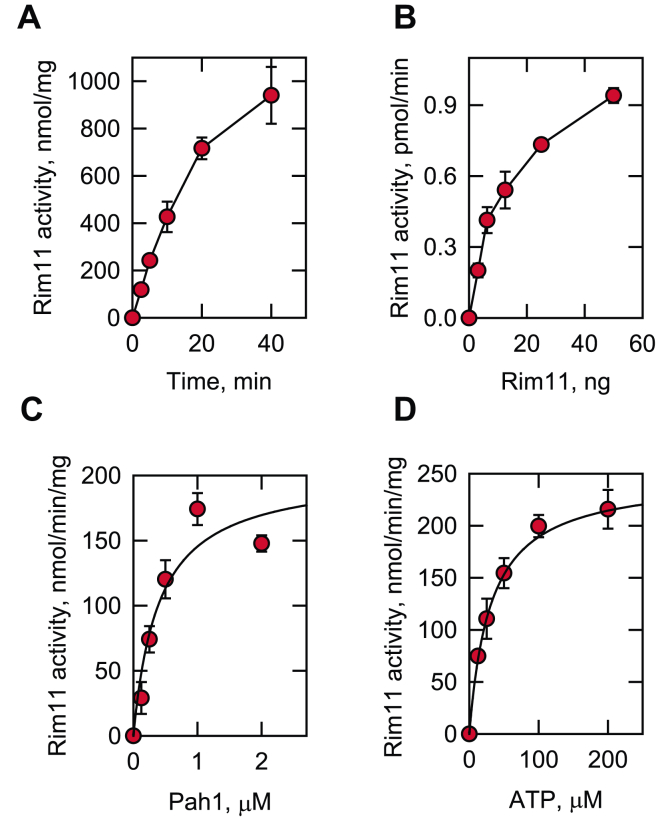
**Rim11 activity on Pah1 is dependent on the reaction time and the amounts of Rim11, Pah1, and ATP.** The *E. coli*-expressed Pah1 was incubated at 30 °C with Rim11 and [γ-^32^P]ATP, subjected to SDS-PAGE, and analyzed by phosphorimaging. The enzyme reaction was conducted by varying the reaction time (*A*) and the amounts of Rim11 (*B*), Pah1 (*C*), and ATP (*D*). *A*, 0.25 μM Pah1/100 μM ATP/5 ng Rim11; *B*, 0.25 μM Pah1/100 μM ATP/10 min; *C*, 100 μM ATP/5 ng Rim11/10 min; *D*, 0.25 μM Pah1/10 min/Rim11. The data shown are means ± SD (*error bars*) from triplicate assays.

The Rim11 protein kinase was further characterized with Pah1 as substrate. The enzyme activity was dependent on reaction time and the amount of Rim11, indicating that the protein kinase follows zero order kinetics (Fig. 6, *A* and *B*). In addition, the Rim11 activity was dependent on the concentrations of Pah1 and ATP (Fig. 6, *C* and *D*). The *V*_max_ and *K*_*m*_ values of Rim11 for Pah1 were 169 units/mg and 0.4 μM, respectively, and those for ATP were 253 units/mg and 30 μM, respectively. At the point of maximum phosphorylation, Rim11 catalyzed the incorporation of 2 mol of phosphate/mol of Pah1. Overall, these enzymological properties demonstrate that Pah1 is a *bona fide* substrate of Rim11.

### Phosphorylation of Pah1 by Rim11 inhibits its PAP activity

**Figure 7:**
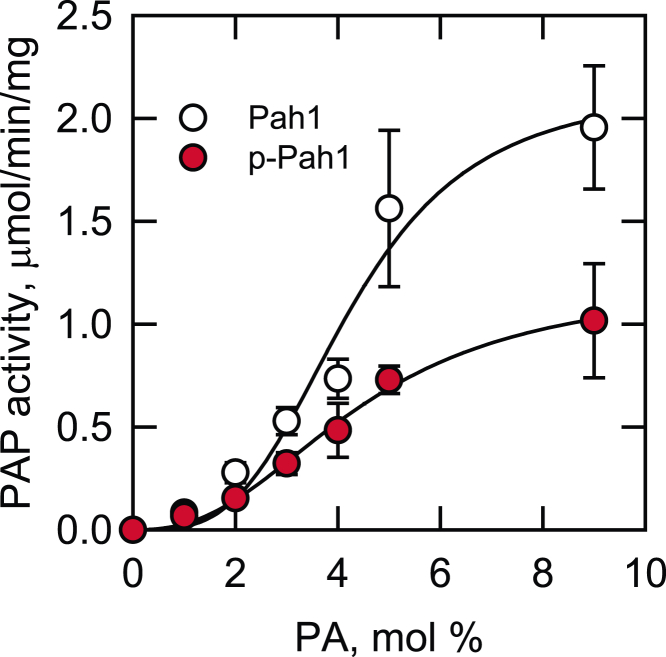
**Phosphorylation of Pah1 by Rim11 inhibits PAP activity.** The *E. coli*-expressed Pah1 (0.5 μg) was phosphorylated by Rim11 (0.2 μg) for 2 h with 100 μM ATP in a total volume of 20 μl. The unphosphorylated Pah1 control was incubated under the same reaction condition in the absence of Rim11. After the incubation, 10% of the reaction mixture was measured for PAP activity. The surface concentration of PA (mol %) was adjusted by maintaining the molar concentration of PA at 0.2 mM and varying the molar concentration of Triton X-100 (76). The data shown are means ± SD (*error bars*) from triplicate assays. PA, phosphatidate; PAP, phosphatidate phosphatase.

We examined the PAP activity of Pah1 phosphorylated by Rim11. The *E. coli*-expressed Pah1 was phosphorylated by Rim11 and then measured for its PAP activity with respect to the surface concentration of PA in Triton X-100/PA-mixed micelles (2, 76). Under this assay condition, PAP activity is independent of the molar concentration of PA (76). As described previously (2), the PAP activity of unphosphorylated Pah1 exhibited positive cooperative kinetics (*n* = 3.4) with respect to PA (Fig. 7). The *V*_max_ and *K*_m_ values of the unphosphorylated enzyme were 2.1 μmol/min/mg and 4.2 mol %, respectively. The phosphorylation of Pah1 by Rim11 caused a decrease in positive cooperative kinetics (*n* = 2.5) and PAP activity with the inhibitory effect stronger at the higher concentrations of PA. The *V*_max_ value (1.2 μmol/min/mg) of the phosphorylated Pah1 was 1.8-fold lower than that of its unphosphorylated form. However, the *K*_m_ value (4.5 mol %) of the phosphorylated enzyme for the substrate PA was not significantly different from that of the unphosphorylated form. These kinetic data indicate that the Rim11-mediated inhibition of Pah1 PAP occurs by decreasing its catalytic efficiency.

### Prephosphorylation of Pah1 by Pho85-Pho80 increases its phosphorylation by Rim11

**Figure 8:**
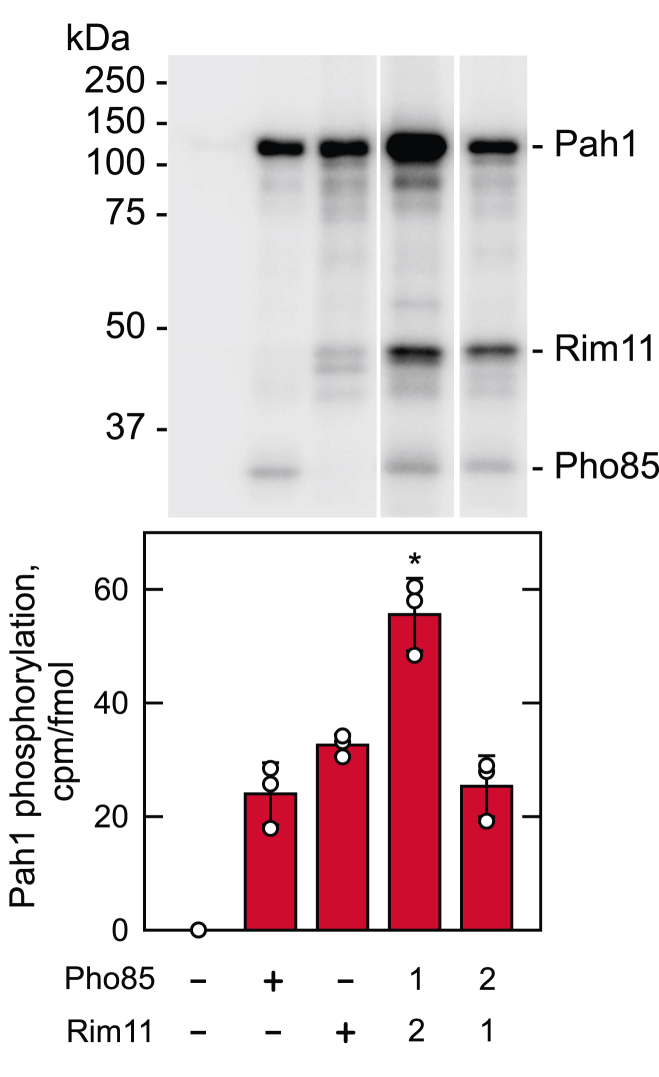
**Prephosphorylation of Pah1 by Pho85-Pho80 increases phosphorylation by Rim11**. The *E. coli*-expressed Pah1 (0.2 μg) was incubated with nonradioactive ATP (200 μM) in the absence or presence of Pho85-Pho80 (0.25 μg) and Rim11 (0.1 μg) for 2 h at 30 °C. Following the incubation, [γ-^32^P]ATP (3000 Ci/mmol) was added to the reaction mixture to adjust the final specific radioactivity to 5000 cpm/pmol. Pah1 prephosphorylated by Pho85-Pho80 and Rim11 and was then incubated for 1 h with Rim11 (100 ng) and Pho85-Pho80 (250 ng), respectively. The order of the phosphorylations is indicated by the numbers *1* and *2*. The ^32^P-labeled Pah1 was resolved by SDS-PAGE and subjected to phosphorimaging and ImageQuant analysis. The phosphorimage (*upper*) is representative of three experiments, whereas the bar chart (*lower*) shows the individual data points and the quantification of the three experiments ± SD (*error bars*). The positions of phosphorylated Pah1, Rim11, and Pho85 are indicated with molecular mass standards. ∗, *p* < 0.05 *versus* the other conditions of phosphorylation.

Phosphorylation of substrates by mammalian GSK3β typically requires a priming phosphorylation at the C-terminal Ser/Thr residue in the motif S/T*XXX*S/T by another protein kinase (77, 78). Although Pah1 was phosphorylated by Rim11 without the priming phosphorylation, its phosphorylation sites Ser12 and Thr164 are in the consensus motif. As Ser168 is a target site of Pho85-Pho80 (47) (Fig. 2), we questioned whether Pah1 phosphorylation by Pho85-Pho80 affects its phosphorylation by Rim11. To examine the prephosphorylation effect, the *E. coli*-expressed (*i.e.*, unphosphorylated) Pah1 was phosphorylated first by Pho85-Pho80 with nonradioactive ATP and then by Rim11 with [γ-^32^P]ATP. The measurement of radioactive Pah1 showed that its phosphorylation by Pho85-Pho80 stimulated (∼2-fold) the subsequent phosphorylation by Rim11 (Fig. 8). In the reciprocal experiment, however, Pah1 phosphorylation by Rim11 did not affect its subsequent phosphorylation by Pho85-Pho80 (Fig. 8). In this analysis, we also noted that Rim11, which autophosphorylates on Tyr199 (71, 75), was phosphorylated by Pho85-Pho80 (Fig. 8); phosphoamino acid analysis indicated that the phosphorylation occurs on the serine residue.

### Nem1-Spo7 protein phosphatase dephosphorylates Pah1 phosphorylated by Rim11

**Figure 9:**
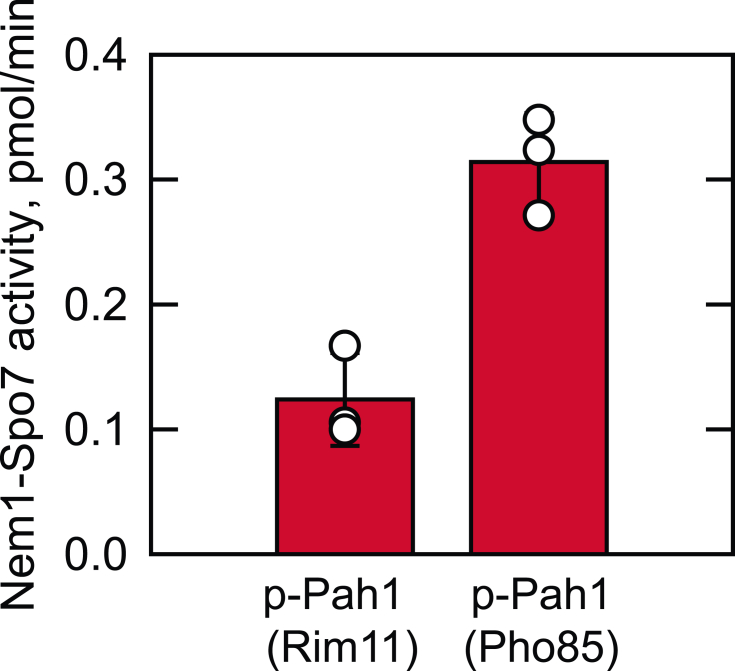
**Nem1-Spo7 utilizes Rim11-phosphorylated Pah1 as a substrate.** The *E. coli*-expressed Pah1 was phosphorylated by Rim11 or Pho85-Pho80 with [γ-^32^P]ATP. The ^32^P-labeled Pah1 was purified and used as a substrate for protein phosphatase activity of Nem1-Spo7 reconstituted in phospholipid vesicles. The individual data points are also shown. The data are means ± SD (*error bars*) from triplicate assays.

The Nem1-Spo7 protein phosphatase (53) is responsible for the dephosphorylation of Pah1 at the nuclear/ER membrane (34, 54, 55) (Fig. 1). We examined whether the phosphatase complex dephosphorylates Pah1 phosphorylated by Rim11. For this analysis, the *E. coli*-expressed Pah1 was phosphorylated by Rim11 with [γ-^32^P]ATP to synthesize a radiolabeled substrate. The ^32^P-labeled Pah1 was mixed with the Nem1-Spo7 complex reconstituted in phospholipid vesicles, and the phosphatase activity was scored by the release of ^32^P_i_ from the radioactive protein (79). The protein phosphatase was active on the Rim11-phosphorylated Pah1, releasing radioactive phosphate from the substrate (Fig. 9). As described previously (79), Nem1-Spo7 catalyzed the dephosphorylation of the Pho85-Pho80-phosphorylated Pah1 (Fig. 9). When compared, the Nem1-Spo7 phosphatase activity on the Rim11-phosphorylated Pah1 was 2.5-fold lower than that on the Pho85-Pho80-phosphorylated protein.

### Rim11 contributes to the Nem1-Spo7-mediated regulation of Pah1 for TAG synthesis

**Figure 10:**
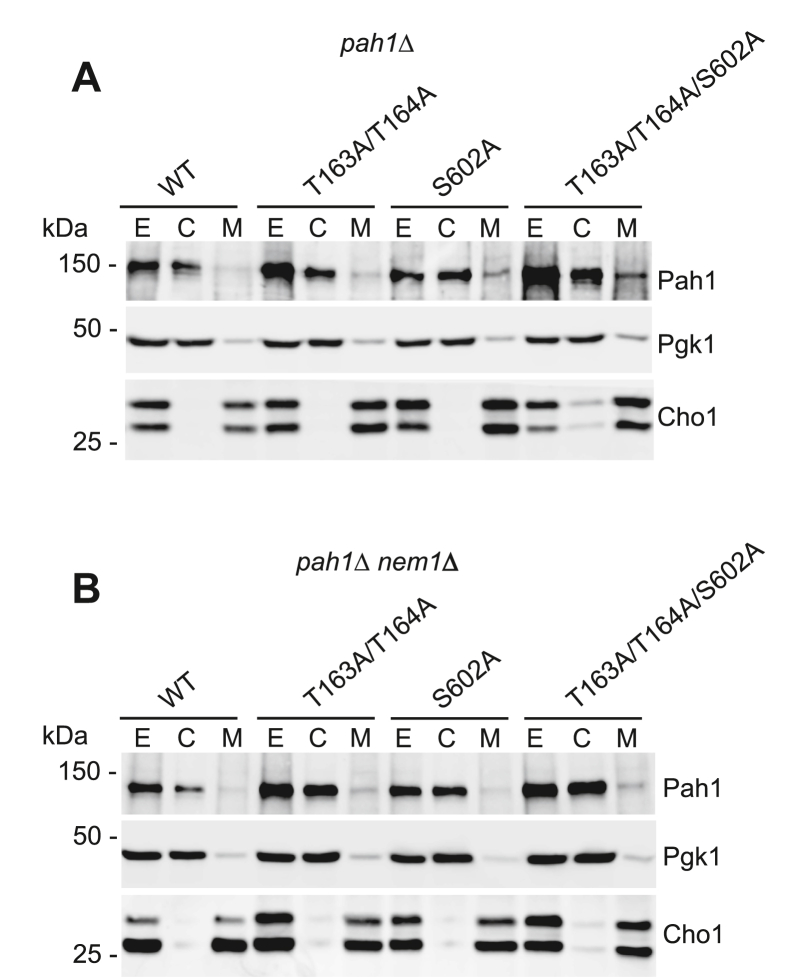
**Subcellular localization of Pah1 mutants lacking phosphorylation sites for Rim11.** The *pah1*Δ (*A*) and *pah1*Δ *nem1*Δ (*B*) cells expressing the indicated wildtype and Rim11 phosphorylation-deficient forms of Pah1 were grown at 30 °C to the mid-logarithmic phase in synthetic complete–Leu medium. Cell extracts (*E*) were fractionated into the cytosolic (*C*) and membrane (*M*) fractions by centrifugation at 100,000*g* for 1 h at 4 °C. The membrane fraction was resuspended in the same volume as the cytosolic fraction, and equal volumes of the fractions were subjected to SDS-PAGE using a 10% polyacrylamide gel, followed by immunoblot analysis using anti-Pah1, anti-Pgk1 (cytosol marker), and anti-Cho1 (ER marker) antibodies. The positions of Pah1, Pgk1, and Cho1 (the upper band showing slower electrophoretic migration is a phosphorylated form of the protein (109)) are indicated with molecular mass standards. The data shown are representative of three experiments.

We constructed the *rim11*Δ mutation to examine the effect of Rim11 deficiency on the phosphorylation of Pah1 and its function in TAG synthesis. However, the slower growth of the *rim11*Δ mutant made it difficult to interpret the lipid synthesis, which is dependent on the growth state (11, 13), in a meaningful way. Accordingly, we constructed the alanine mutations for the major sites (*e.g.*, Thr163, Thr164, and Ser602) of Pah1 phosphorylation by Rim11 and examined the physiological consequences of the phosphorylation deficiency. The phosphorylation-deficient alleles of *PAH1* (T163A/T164A, S602A, and T163A/T164A/S602A) were expressed on a low-copy plasmid in the *pah1*Δ and *pah1*Δ *nem1*Δ strains. The *pah1*Δ *nem1*Δ mutant, which lacks the Nem1 protein phosphatase catalytic subunit (14, 53), was used to examine the dependency of Pah1 function on the Nem1-Spo7 protein phosphatase complex (48). The *nem1*Δ mutation also affords the analysis of the phosphorylation-deficient Pah1 in the presence of phosphorylation on the nonmutated sites (34, 48). The level of Pah1 was examined in the exponential-phase cells because the protein is subject to proteasome-mediated degradation in the stationary phase (63). Western blot analysis of cell extracts with anti-Pah1 antibody confirmed that the phosphorylation-deficient Pah1 mutants are expressed in both genetic backgrounds and that the mutations do not have a major effect on the amount of Pah1 (Fig. 10, *A* and *B*). In addition, we examined whether the localization of Pah1 is affected by the lack of its phosphorylation by Rim11. As described previously (80), the wildtype Pah1 was found in the cytosolic and membrane fractions of the cell with more than 90% of the enzyme in the cytosol; the Rim11 phosphorylation-deficient mutations did not have a major effect on the localization of Pah1 (Fig. 10).

**Figure 11:**
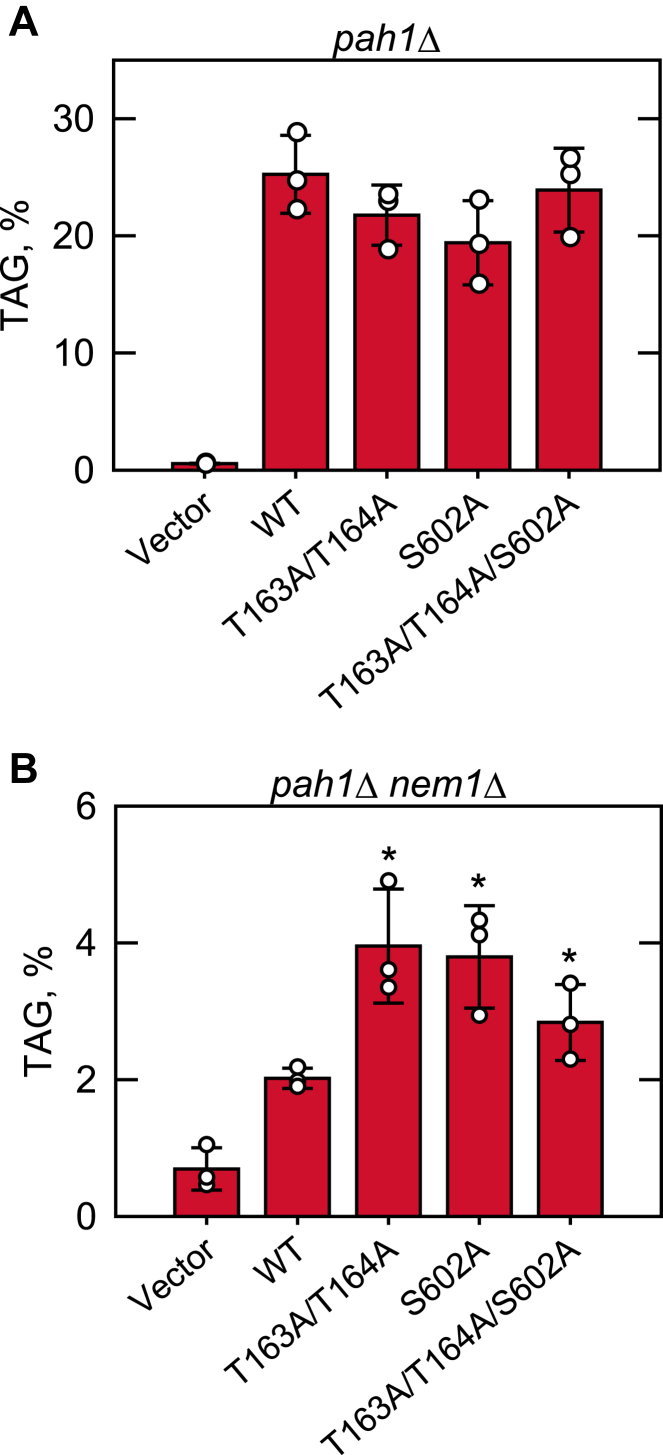
**Rim11 phosphorylation-deficient mutations in Pah1 cause an increase in TAG content in cells lacking the Nem1-Spo7 protein phosphatase complex.** The *pah1*Δ (*A*) and *pah1*Δ *nem1*Δ (*B*) cells expressing wildtype and Rim11 phosphorylation-deficient forms of Pah1 were grown at 30 °C to the stationary phase in 5 ml of synthetic complete–Leu medium containing [2-^14^C]acetate (1 μCi/ml). Lipids were extracted, separated by one-dimensional TLC, and subjected to phosphorimaging, followed by ImageQuant analysis. The percentage shown for TAG was normalized to the total ^14^C-labeled chloroform-soluble fraction. The data are means ± SD (*error bars*) from three separate experiments. The individual data points are also shown. ∗, *p* < 0.05 *versus* WT. TAG, triacylglycerol.

As the effect of Pah1 PAP activity on TAG content is pronounced in stationary phase cells (2, 11, 17), our experiments were performed at this phase of growth. The *pah1*Δ and *pah1*Δ *nem1*Δ cells expressing the phosphorylation-deficient forms of Pah1 were labeled with [2-^14^C]acetate, followed by the extraction and analysis of TAG content. In *pah1*Δ cells expressing wildtype Pah1, TAG accounted for 25% of the total ^14^C-labeled lipids (Fig. 11*A*). The TAG content was not significantly affected by the Rim11 phosphorylation-deficient mutations. When wildtype Pah1 was expressed in *pah1*Δ *nem1*Δ cells, TAG accounted for only 2% of the total lipids (Fig. 11*B*). This result confirms previous work showing that the dephosphorylation of Pah1 by the Nem1-Spo7 protein phosphatase complex is crucial for its function in TAG synthesis (34, 80). The TAG content of *pah1*Δ *nem1*Δ cells expressing the Rim11 phosphorylation-deficient mutant forms of Pah1 was 1.4- to 2-fold higher when compared with that of the cells expressing the wildtype Pah1 (Fig. 11*B*). This result indicates that the lack of Pah1 phosphorylation on Thr163, Thr164, and Ser602 partially bypasses the requirement of the Nem1-Spo7 complex for its localization to the membrane.

### Rim11 levels during cell growth

**Figure 12:**
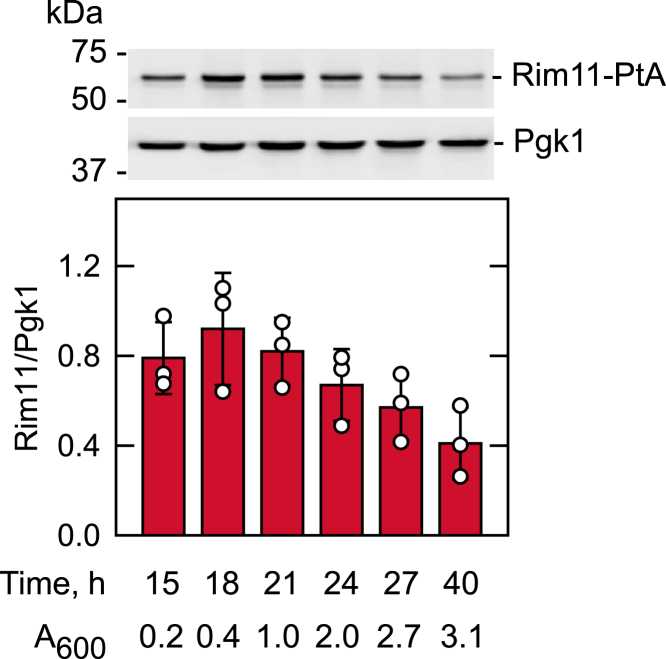
**Rim11 levels during cell growth.** Yeast cells expressing TAP-tagged Rim11 were grown for the indicated time intervals in synthetic complete medium. The cell density measured by *A*_600_ at the time of harvest is also indicated. Cell extracts were prepared, and 25 μg total protein was used for Western blot analysis with anti-protein A and anti-Pgk1 (loading control) antibodies. *Top*, portions of representative blots from three independent experiments are shown, and the positions of molecular mass standards, Rim11-PtA, and Pgk1 are indicated. *Bottom*, amounts of Rim11-PtA and Pgk1 were quantified with ImageQuant software, and the relative amount of Rim11-PtA/Pgk1 is presented. The data shown in the figure are the average of three independent experiments ± SD (*error bars*). The individual data points are also shown.

To better understand the role of Rim11 protein kinase, we examined its protein level during cell growth. Yeast cells expressing the TAP-tagged chromosomal *RIM11* were grown in SC medium from the exponential to the stationary phase, and the cell extracts were analyzed for Rim11 abundance by Western blotting with anti-protein A antibody (Fig. 12). The level of Rim11 was higher in the exponential phase of growth and reduced as the cells progressed into the stationary phase. The amount of Rim11 at its maximum abundance in the exponential phase (18 h) was ∼ 2-fold higher when compared with that in the stationary phase (40 h).

## Discussion

Rim11 was originally identified as a protein being required for signal transduction during entry into meiosis (72). It promotes the formation of the Ime1-Ume6 transcriptional activator complex by phosphorylating the protein subunits (73, 74). In addition, the protein kinase was shown to be active on 71 proteins including Pah1 in the global analysis of protein kinase substrates (70). In this work, we confirmed that Pah1 is a *bona fide* substrate for Rim11, identified the Rim11-mediated phosphorylation sites in Pah1, showed that Rim11 is maximally expressed in the exponential phase of growth, and that Rim11 plays a regulatory role in lipid synthesis by controlling the phosphorylation state of Pah1 during vegetative cell growth.

In the enzymological properties of Rim11, its *K*_m_ value for Pah1 is in the range of the values reported for casein kinases I (52) and II (51), cyclin-dependent protein kinases Cdc28 (48) and Pho85 (47), and protein kinases A (49) and C (50) (Table 1). In contrast, its *K*_m_ for ATP is 5- to 12-fold higher than that of the other protein kinases (Table 1), suggesting that Rim11 activity on Pah1 is relatively lower when the cellular concentration of ATP is reduced (*e.g.*, < 30 μM). Considering that one protein kinase is not sufficient to properly phosphorylate Pah1, the employment of multiple protein kinases provides a mechanism to ensure its phosphorylation for the Nem1-Spo7-dependent regulation. In this regard, Ser602 is shown to be a site whose phosphorylation is secured by Rim11, casein kinase I (52), Pho85-Pho80 (47), and Cdc28-cyclin B (48) (Fig. 2).

**Table 1:** Kinetic properties of protein kinases that phosphorylate Pah1

Protein kinase	Pah1 *K*_*m*_	ATP *K*_*m*_	Stoichiometry	Reference
	*μM*	*μM*	*mol phosphate/mol Pah1*	
Rim11	0.40	30	2.0	This study
Casein kinase I	0.21	2.4	2.8	(52)
Casein kinase II	0.23	5.5	2.0	(51)
Cdc28-cyclin B	0.21	5.8	0.8	(48)
Pho85-Pho80	0.25	3.7	4.0	(47)
Protein kinase A	0.44	4.4	1.0	(49)
Protein kinase C	0.75	4.5	0.8	(50)

Of six sites of Pah1 phosphorylated by Rim11, only Ser12 and Thr164 are contained within the canonical GSK3β substrate recognition motif S/T*XXX*S/T (77, 78), in which the phosphorylation of the N-terminal Ser/Thr residue requires the phosphorylation of the C-terminal Ser/Thr residue at +4 position (77, 78). It is obvious that the phosphorylation of Pah1 on Ser602, which accounts for 64% of the total phosphorylation, does not require its prephosphorylation. Yet, the protein prephosphorylation by Pho85-Pho80 causes an ∼ 2-fold increase in the subsequent phosphorylation by Rim11. This stimulatory effect may result from the priming phosphorylation of Pah1 on Ser168 by Pho85-Pho80 (47) (Fig. 2). Alternatively, the prephosphorylation of Pah1 may simply stimulate its subsequent phosphorylation by Rim11. Unlike Ser168, Ser16, the putative priming site for Ser12, has not been identified as a phosphorylation site.

Parenthetically, there are 48 putative target sites for GSK3β in Pah1, and Rim11 is not the only GSK3β homolog in *S. cerevisiae*. The other GSK3β homologs include Mck1 (81), Mrk1 (82), and Ygk3 (83). Rim11 and Mck1are paralogs of Mrk1 and Ygk3, respectively, which arose from gene duplication. In the global analysis of protein phosphorylation, Pah1 is a substrate of Mck1 but not of Ygk3 (70). Pah1 is not the first lipid metabolic enzyme known to be regulated by a GSK3β homolog in yeast. For example, the Elo2 fatty acid elongase is stimulated by Mck1 and to a lesser extent by Rim11 for the synthesis of very-long-chain fatty acids (84), which are precursors to yeast sphingolipids (85, 86). The phosphorylation effect on Elo2 is shown to be in the protection of the enzyme against its proteolytic degradation (84).

The Rim11-mediated phosphorylation of Pah1 exhibits an inhibitory effect on its PAP activity. According to the kinetic parameters of Pah1 PAP, the inhibitory effect is on the catalytic efficiency, but not on the affinity for PA. The inhibitory effect of Pah1 phosphorylation is also shown by Pho85-Pho80 (47), protein kinase A (47), and casein kinase II (51). In contrast, casein kinase I (52) and protein kinase C (50) stimulate Pah1 for its PAP activity. The phosphorylation of Pah1 is required for its translocation to the nuclear/ER membrane *via* the Nem1-Spo7 protein phosphatase complex (69). The major sites of the protein responsible for this regulation are the seven sites phosphorylated by Pho85-Pho80 (34, 47, 48), and the phosphorylated form of the sites serves as the most optimum substrate for the Nem1-Spo7 protein phosphatase (79). When compared as a substrate, Pah1 phosphorylated by Rim11 is not as good as that phosphorylated by Pho85-Pho80 but better than that phosphorylated by other protein kinases. Of the known protein kinase–phosphorylation site relationships in Pah1 (47, 48, 49, 50, 51), the activity of the Nem1-Spo7 protein phosphatase is in the order of the sites phosphorylated by Pho85-Pho80 (100%) > Rim11 (38%) > protein kinase A (25%) = CKII (25%) > Cdc28-cyclin B (15%) > PKC (7.5%) (51).

Rim11 phosphorylates Pah1 mainly on Ser602, which is one of the seven sites required for its membrane translocation by Nem1-Spo7 protein phosphatase complex. Accordingly, Pah1 deficient in the phosphorylation site bypasses the requirement of Nem1-Spo7 as indicated by its partial functional role in the *nem1*Δ mutant for TAG synthesis. In this work, the Rim11-specific sites T163 and T164 were also identified to be important for the Nem1-Spo7-mediated control of Pah1 function. Interestingly, the combination of S602A and T163A/T164A does not show an additive mutational effect, suggesting that phosphorylations on those sites are redundant in the Nem1-Spo7-dependent control of Pah1. Pah1 phosphorylation by multiple protein kinases is considered important to better control its translocation level that is required differently during growth from the exponential to the stationary phase. Whether other GSK3β homologs (Mck1, Mrk1, and Ygk3) are involved in the control of Pah1 function is a question that needs to be addressed to better understand its regulation by phosphorylation.

## Experimental procedures

### Reagents

Chemicals were reagent grade. Lipids and silica gel GHL TLC plates, respectively, were from Avanti Polar Lipids and Analtech. Bio-Rad was the supplier of DNA size ladders, molecular mass protein standards, and reagents for electrophoresis and Western blotting. Cayman Chemical was the source of leupeptin and pepstatin. Clontech supplied carrier DNA for yeast transformations. Growth media were obtained from Difco Laboratories. Expedeon was the source of InstantBlue (Coomassie blue) protein stain. GE Healthcare was the source of Q-Sepharose, IgG-Sepharose, PVDF membrane, and the chemifluorescence Western blotting detection kit. Invitrogen supplied mouse anti-phosphoglycerate kinase antibodies (product number: 459250; lot number: E1161). Millipore Sigma was the source of ATP, bovine serum albumin, cellulose TLC plates, chymotrypsin, phosphoamino acid standards, protease and phosphatase inhibitors, Ponceau S stain, L-1-tosylamido-2-phenylethyl chloromethyl ketone-trypsin, Triton X-100, rabbit anti-calmodulin binding protein epitope tag antibody (product no. 07-482, lot no. 3467112), rabbit anti-protein A antibody (product no. P3775, lot no. 053M4806V), and alkaline phosphatase–conjugated goat anti-mouse IgG antibodies (product no. A3562; lot no. SLBG1482V). Scintillation counting supplies were from National Diagnostics. Q5 site-directed mutagenesis kit and other reagents for DNA manipulations were from New England Biolabs. Radiochemicals were purchased from PerkinElmer Life Sciences. Qiagen supplied DNA gel extraction and plasmid purification kits and the nickel-nitrilotriacetic acid agarose resin. Pierce mass spectrometry grade proteases and strong anion exchange spin columns, alkaline phosphatase–conjugated goat anti-rabbit IgG antibody (product no. 31340, lot number: NJ178812), and *S. cerevisiae* strain BY4741-*RIM11*-TAP were purchased from Thermo Fisher Scientific. Phos-tag Acrylamide AAL-107 was purchased from Wako Chemicals.

### *Strains*, *plasmids*, *and growth* conditions

The yeast and bacterial strains used in this study are listed in Table 2. *S. cerevisiae* strain W303-1A was the source of the *RIM11* gene and used for the construction of the *rim11*Δ mutant SKY001 and for the expression of plasmid-borne *RIM11*. BY4741-*RIM11*-TAP was the source of the *RIM11*-TAP gene that was cloned into pYES2, a galactose-inducible overexpression plasmid, for the construction of pSK001. *E. coli* strains DH5α and BL21(DE3)pLysS were used for the propagation of plasmids and heterologous expression of His_6_-tagged Pah1, respectively.

**Table 2:** Strains and plasmids used in this study

Strain or plasmid	Genotype or relevant characteristics	Source or Reference
**Strain**		
***S*. *cerevisiae***		
RS453	*MAT***a** *ade2-1 his3-11,15 leu2-3,112 trp1-1 ura3-52*	(110)
SS1026	*pah1*Δ*::TRP1* derivative of RS453	(14)
SS1132	*pah1*Δ*::TRP1 nem1*Δ*::HIS3* derivative of RS453	(48)
W303-1A	*MAT***a***ade2-1 can1-100 his3-11,15 leu2-3,112 trp1-1 ura3-1*	(111)
BY4741	*MAT***a***his3*Δ*1 leu2*Δ*0 met15*Δ*0 ura3*Δ*0*	Thermo Fisher Scientific
BY4741-*rim11*Δ*::KanMX*	*rim11*Δ*::KanMX* of BY4741	Thermo Fisher Scientific
SKY001	*rim11*Δ*::KanMX* derivative of W303-1A	This study
BY4741-*RIM11*-TAP	TAP-tagged *RIM11* strain	Thermo Fisher Scientific
***E. coli***		
DH5α	F^-^ φ80 *lacZ*ΔΜ15Δ (*lacZYA*-*argF*)U169 *deoR rec*A1 *end*A1 *hsd*R17(*r*_k_^-^*m*_*k*_^+^) *pho*A *sup*E44 λ^−^*thi-*1 *gyr*A96 *rel*A1	(88)
BL21(DE3)pLysS	F^-^*ompT hsdS*_*B*_ (*r*_*B*_^-^*m*_*B*_^-^) *gal dcm* (DE3) pLysS	Novagen
BL21(DE3)	F^-^*ompT hsdS*_*B*_ (*r*_*B*_^-^*m*_*B*_^-^) *gal dcm* (DE3)	Invitrogen
**Plasmid**		
pET-15b	*E. coli* expression vector with N-terminal His_6_-tag fusion	Novagen
pGH313	*PAH1* coding sequence inserted into pET-15b	(2)
pRS415	Single-copy number *E. coli*/yeast shuttle vector with *LEU2*	(112)
pGH315	*PAH1* inserted into pRS415	(80)
pSK005	*PAH1* (T163A/T164A) derivative of pGH315	This study
pSK006	*PAH1* (S602A) derivative of pGH315	This study
pSK007	*PAH1* (T163A/T164A/S602A) derivative of pGH315	This study
pHC204	*PAH1* (S110A/S114A/S168A/S602A/T723A/S744A/S748A) derivative of pGH315	(80)
pSK003	*RIM11* inserted into pRS415	This study
pYES2	High-copy number *E. coli*/yeast shuttle vector with *URA3* and the *GAL1* promoter	Thermo Fisher Scientific
pSK001	*RIM11*-TAP in pYES2	This study
EB1164	*PHO85-*His_6_ derivative of pQE-60	(96)
EB1076	*PHO80* derivative of pSBETA	(96)
YCplac111-*GAL1/10*- *NEM1*-PtA	*NEM1*-PtA under control of *GAL1/10* promoter inserted in *CEN/LEU2* plasmid	(14)
pRS313-*GAL1/10*-*SPO7*	*SPO7* under control of *GAL1/10* promoter inserted in *CEN/HIS3* plasmid	(24)

The plasmids used in this study are listed in Table 2. Plasmids containing *PAH1* (T163A/T164A), *PAH1* (S602A), and *PAH1* (T163A/T164A/S602A) were generated from pGH315 using the Q5 Site-directed mutagenesis kit. The *RIM11* gene was amplified by PCR with the addition of NotI/XhoI sites from the W303-1A genomic DNA and inserted into the NotI/XhoI sites of plasmid pRS415 to construct pSK003. Plasmid pSK001 was generated by inserting the *RIM11*-TAP sequence, which was PCR amplified from BY4741-*RIM11*-TAP DNA, into pYES2 at the KpnI/NotI sites. The *rim11*Δ mutant strain SKY001 was constructed from W303-1A by homologous recombination of the *rim11*Δ*::KanMX* cassette that was PCR amplified from the yeast mutant collection. Standard methods were used for isolation of chromosomal and plasmid DNA, for digestion and ligation of DNA, and for PCR amplification of DNA (87, 88, 89). Plasmid transformations of yeast (90) and *E*. *coli* (88) were performed by standard methods. DNA constructs were confirmed by PCR analysis and DNA sequencing.

Yeast cells were cultured using standard methods (87, 88). Solid media plates contained 2 or 1.5% agar, respectively, for the growth of yeast or bacteria. Yeast and bacterial growth in liquid medium was monitored by absorbance at 600 nm (*A*_600_) using a spectrophotometer. Yeast cells carrying a plasmid were grown at 30 °C in synthetic drop-out medium, which lacks a specific amino acid from synthetic complete (SC) medium for the plasmid selection. Cells of strain W303-1A harboring plasmid pSK001 were inoculated into 250 ml of SC-Ura/2% glucose medium to a final cell density of *A*_600_ ∼ 0.1 and then grown to saturation. The saturated culture was harvested by centrifugation at 1500*g* for 10 min and the cell pellet was resuspended (*A*_600_ ∼ 0.4) in 2 L of SC-Ura/1% raffinose/2% galactose medium and incubated for 14 h (*A*_600_ ∼ 1.0) with shaking at 250 rpm. The *E*. *coli* cells were grown at 37 °C in lysogeny broth (LB) medium (1% tryptone, 0.5% yeast extract, 1% NaCl, pH 7.0); ampicillin (100 μg/ml) was added to select for cells carrying plasmids. For His_6_-tagged Pah1 expression, the bacterial cells harboring plasmid pGH313 were grown to *A*_600_ ∼ 0.5 at room temperature in 500 ml of LB medium containing ampicillin (100 μg/ml) and chloramphenicol (34 μg/ml); expression was induced for 1 h with 1 mM isopropyl-*β*-D-thiogalactoside (2).

### Lipid labeling and analysis

Yeast cells were labeled to steady state with [2-^14^C]acetate (91); the lipids were extracted from the cells by the method of Bligh and Dyer (92) as described by Fakas *et al*. (93). Neutral lipids were resolved by one-dimensional TLC on silica gel plates using the solvent system hexane/diethyl either/glacial acetic acid (40:10:1, v/v) (94). The resolved lipids were visualized by phosphorimaging with a Storm 860 Molecular Imager (GE Healthcare) and quantified by ImageQuant software using a standard curve of [2-^14^C]acetate. The identity of radiolabeled TAG was confirmed by comparison with the migration of authentic standard visualized by staining with iodine vapor.

### Preparation of yeast cell extracts and subcellular fractions

#### Cell extracts

All steps were performed at 4 °C. Yeast cultures were harvested by centrifugation at 1500*g* for 5 min. The collected cells were washed with water and resuspended in 50 mM Tris-HCl (pH 7.5) buffer containing 0.3 M sucrose, 10 mM 2-mercaptoethanol, 1 mM EDTA, 0.5 mM phenylmethylsulfonyl fluoride, 1 mM benzamidine, 5 μg/ml aprotinin, 5 μg/ml leupeptin, and 5 μg/ml pepstatin. Glass beads (0.5 mm diameter) were added to cell suspensions and then subjected to five repeats of 1-min burst and 2-min cooling using a BioSpec Products Mini-Beadbeater-16 (95). The disrupted cells were centrifuged at 1500*g* for 10 min to separate unbroken cells and cell debris (pellet) from cell extracts (supernatant). The cell extract was centrifuged at 100,000*g* for 1 h to separate the cytosol (supernatant) from the membrane (pellet). The membrane fraction was resuspended in the same volume of cell disruption buffer.

### Purification of enzymes

#### Purification of Pah1

The His_6_-tagged Pah1 expressed in *E. coli* was purified from cell extracts by affinity chromatography with nickel-nitrilotriacetic acid-agarose (2), followed by ion exchange chromatography with Q-Sepharose (79). *S. cerevisiae*-expressed TAP-tagged Pah1 was purified from cell extracts by IgG-Sepharose affinity chromatography, anion exchange chromatography, and size exclusion chromatography (61); purified preparations were stored at – 80 °C.

#### Purification of Rim11

All steps were performed at 4 °C unless otherwise indicated. The galactose-induced yeast culture of W303-1A expressing the TAP-tagged Rim11 was harvested, and the cell pellet was resuspended in 16 ml of 50 mM Tris-HCl (pH 8.0) buffer containing 150 mM NaCl, 1 mM EDTA, and Roche EDTA-free protease inhibitors and lysed with glass beads using a Mini-Beadbeater-16 (5 repeats of 1-min burst with 2-min cooling between bursts). The cell lysate was centrifuged at 1500*g* for 10 min, and the supernatant was mixed with an equal volume of 50 mM Tris-HCl (pH 8.0) buffer containing 150 mM NaCl, 1 mM EDTA, Roche EDTA-free protease inhibitors, and 2% Triton X-100 and centrifuged at 100,000*g* for 1 h. The supernatant was applied to a 0.5-ml IgG-Sepharose column equilibrated with 50 mM Tris-HCl (pH 8.0) buffer containing 150 mM NaCl, 0.5 mM EDTA, and 0.1% Triton X-100. The column was washed with 20 column volumes of the equilibration buffer and then incubated at room temperature for 1 h with 75 units of tobacco etch virus protease in 0.5 ml 50 mM Tris-HCl (pH 8.0) buffer containing 150 mM NaCl, 0.5 mM EDTA, 0.1% Triton X-100, 1 mM dithiothreitol. The protein A–free Rim11 was eluted from the IgG-Sepharose resin with the chromatography buffer. The tobacco etch virus protease was removed from the Rim11 preparation by passage through a strong anion exchange spin column equilibrated with 20 mM Tris-HCl (pH 8.0) buffer containing 150 mM NaCl and 10% glycerol. Rim11 was then eluted from the spin column by increasing the NaCl concentration in the equilibration buffer to 250 mM. Purified Rim11 preparations were stored at −80 °C.

#### Purification of the Pho85-Pho80 protein kinase complex

The His_6_-tagged Pho85-Pho80 complex was purified from *E. coli* BL21(DE3) cells that expressed plasmids EB1164 and EB1076 by affinity chromatography with nickel-nitrilotriacetic acid-agarose (96).

#### Purification and reconstitution of the Nem1-Spo7 protein phosphatase complex

The protein A–tagged Nem1-Spo7 complex was purified from *S. cerevisiae* strain RS453 expressing plasmids YCplac111-*GAL1/10*-*NEM1*-PtA and pRS313-*GAL1/10*-*SPO7* by affinity chromatography with IgG-Sepharose as described by Siniossoglou *et al.* (97) with the modifications of Su *et al.* (79). The purified complex (9.5 μg) was reconstituted into unilamellar phospholipid vesicles made of phosphatidylcholine/phosphatidylethanolamine/phosphatidylinositol/phosphatidylserine/PA (33.75:22.5:22.5:11.25:10 mol%) by size exclusion chromatography with Sephadex G-50 in 25 mM Tris-HCl (pH 8.0), 250 mM NaCl, and 10 mM 2-mercaptoethanol (98, 99).

### Rim11 protein kinase assay

Rim11 protein kinase activity was measured at 30 °C by following the incorporation of radioactive phosphate from [γ-^32^P]ATP into Pah1 in a total volume of 20 μl as described by Choi *et al.* (48). The reaction mixture contained 50 mM Tris-HCl (pH 7.5), 10 mM MgCl_2_, 100 μM [γ-^32^P]ATP (∼3000 cpm/pmol), 0.25 μM Pah1, 2 mM dithiothreitol, and the indicated amount of Rim11. The phosphorylation reaction was terminated by the addition of 6.7 μl 4x Laemmli sample buffer, followed by SDS-PAGE (100) to resolve the ^32^P-labeled Pah1 from radioactive ATP. The phosphorylated Pah1 was visualized by phosphorimaging using a Storm 860 Molecular Imager (GE Healthcare), and the extent of phosphorylation was quantified by ImageQuant software.

### Phosphoamino acid analysis and phosphopeptide mapping

The ^32^P-labeled Pah1 transferred to PVDF membrane was digested with L-1-tosylamido-2-phenylethyl chloromethyl ketone-trypsin for phosphopeptide mapping or hydrolyzed with 6 N HCl at 100 °C for phosphoamino acid analysis (101, 102, 103). The tryptic digests were analyzed on the cellulose TLC plates first by electrophoresis and then by thin-layer chromatography (101, 102, 103). The acid hydrolysates were mixed with standard phosphoamino acids and separated by two-dimensional electrophoresis on cellulose TLC plates. Radioactive phosphopeptides and phosphoamino acids were visualized by phosphorimaging analysis using a Storm 860 Molecular Imager (GE Healthcare). Nonradioactive phosphoamino acid standards were visualized by ninhydrin staining.

### Phosphorylation site analysis of Pah1 and identification Rim11 peptide sequences by LC-MS/MS

The sites phosphorylated on Pah1 by the Rim11 protein kinase were analyzed by LC-MS/MS at the Center for Integrative Proteomics Research at Rutgers University as described by Park *et al.* (61). Details on the digestion of the Rim11-phosphorylated Pah1 in polyacrylamide gel slices with trypsin, chymotrypsin, or Glu-C; analysis of fragments by LC-MS/MS; and database analysis are provided in Table S2. To confirm the identity of Rim11, 0.76 μg protein contained within an SDS polyacrylamide gel slice was digested with trypsin at 37 °C followed by analysis of the digest by LC-MS/MS (61). The raw data and database results for the peptide analyses of Pah1 and Rim11 are deposited in the MassIVE repository.

### SDS-PAGE and immunoblot analysis

Standard procedures were used for SDS-PAGE (100) and Western blotting (104, 105). In some experiments, Phos-tag AAL-107 (20 μM) and MnCl_2_ (100 μM) were added to polyacrylamide gels for analysis of the phosphorylation state of Pah1. The samples for Western blotting were normalized to total protein loading. Protein transfer from polyacrylamide gels to PVDF membrane was monitored by staining with Ponceau S. Rabbit anti-protein A, anti-calmodulin binding peptide, and anti-Pah1 (48) antibodies were used at a final concentration of 2 μg/ml, whereas the final concentration of anti-Cho1 antibody was 0.25 μg/ml. A dilution of 1:5000 was used with the secondary goat anti-rabbit IgG antibody conjugated with alkaline phosphatase. The enhanced chemifluorescence immunoblotting substrate was used to detect immune complexes. Fluorimaging with a Storm 865 Molecular Imager was used to visualize fluorescence signals from immunoblots; image intensities were analyzed by ImageQuant TL software (GE Healthcare). A standard curve ensured that the immunoblot signals were in the linear range of detection.

### PAP assay

PAP activity was measured at 30 °C by following the release of water-soluble ^32^P_i_ from chloroform-soluble [^32^P]PA (10,000 cpm/nmol), which was produced from DAG by DAG kinase with [γ-^32^P]ATP as described by Carman and Lin (95). The reaction mixture in a total volume of 100 μl contained 50 mM Tris-HCl (pH 7.5), 1 mM MgCl_2_, 2 mM Triton X-100, 0.2 mM PA, and enzyme protein. Enzyme assays were performed in triplicate, and all reactions were linear with time and protein concentration.

### Nem1-Spo7 protein phosphatase assay

Nem1-Spo7 protein phosphatase activity was measured by following the release of ^32^P_i_ from [^32^P]Pah1 as described by Su *et al.* (79). The ^32^P-labeled Pah1 was prepared by incubation of the *E. coli*-expressed Pah1 (4.3 μg) and [γ-^32^P]ATP (20,000 cpm/pmol) for 2 h at 30 °C with Rim11 (300 ng) or Pho85-Pho80 (1.7 μg). The protein kinase and nucleotide were removed from the phosphorylated Pah1 by use of a strong anion exchange spin column (79). The protein phosphatase reaction mixture contained 100 mM sodium acetate (pH 5.0), 10 mM MgCl_2_, 1 mM DTT, [^32^P]Pah1, and reconstituted Nem1-Spo7 complex in a total volume of 20 μl.

### Protein determination

Protein concentration, using bovine serum albumin as the standard, was estimated by the protein–dye binding assay of Bradford (106).

### Data analysis

The statistical analysis of data was determined with Microsoft Excel software. The *p* values < 0.05 were taken as a significant difference. The enzyme kinetics module of SigmaPlot software was used to analyze kinetic data.

## Data availability

Raw MS phosphorylation data and database search results for Pah1, along with the Rim11 analysis data are deposited in the MassIVE repository (https://massive.ucsd.edu/ProteoSAFe/static/massive.jsp) with the accession number MSV000088038. All other data are contained within the article or the supporting information.

## Supporting information

This article contains supporting information. This article contains supporting information contained in two Excel documents (113).

## Conflict of interest

The authors declare that they have no conflicts of interest with the contents of this article.
